# Fabrication of Different Microchannels by Adjusting the Extrusion Parameters for Sacrificial Molds

**DOI:** 10.3390/mi10080544

**Published:** 2019-08-17

**Authors:** Wenlai Tang, Hao Liu, Liya Zhu, Jianping Shi, Zongan Li, Nan Xiang, Jiquan Yang

**Affiliations:** 1 School of Electrical and Automation Engineering, Jiangsu Key Laboratory of 3D Printing Equipment and Manufacturing, Nanjing Normal University, Nanjing 210023, China; 2 Nanjing Institute of Intelligent High-end Equipment Industry Co., Ltd., Nanjing 210042, China; 3 School of Mechanical Engineering, Jiangsu Key Laboratory for Design and Manufacture of Micro-Nano Biomedical Instruments, Southeast University, Nanjing 211189, China

**Keywords:** microfluidics, microchannel, 3D printing, fused deposition modelling (FDM), sacrificial mold

## Abstract

Using the 3D printed mold-removal method to fabricate microchannel has become a promising alternative to the conventional soft lithography technique, due to the convenience in printing channel mold and the compatibility with PDMS material. Although having great potential, the use of single filament extruded by fused deposition modeling (FDM) as the sacrificial channel mold has not been elaborately studied. In this paper, we demonstrate the fabrication of microchannels with different structure and size by controllably extruding the sacrificial channel molds. The influences of the main processing parameters including working distance, extrusion amount and printing speed on the printed microchannels are systematically investigated. The results show that, the circular and low-aspect-ratio straight microchannels with different sizes can be fabricated by adjusting the extrusion amounts. The sinusoidal, 3D curved and cross-linked curved microchannels along straight path can be fabricated, either independently or in combination, by the combined control of the working distance, extrusion amount and printing speed. The complex microchannels with different structural features can also be printed along curved serpentine, rectangular serpentine, and spiral paths. This paper presents a simple and powerful method to fabricate the complex microchannels with different structure and size by just controlling the processing parameters for extruding channel molds.

## 1. Introduction

Microfluidics [1], which can offer the controlled manipulation of fluids and particles at the micro domain, has been widely used in biological [2], medical [3], chemical [4] and environmental [5] fields, due to the advantages of low sample consumption, short processing time and high detection accuracy. Various techniques including photolithography [6], hot embossing [7], laser ablation [8] and most commonly, soft lithography [9] have been utilized to fabricate the microfluidic device with micron-scale channel network. The popularity of soft lithography in fabricating microchannel benefits from the excellent performance of polydimethylsiloxane (PDMS), which is relatively cheap, gas permeable, transparent and suitable for rapid prototyping [10].

However, the soft lithography technique generally involves a series of processes such as UV-photolithography, PDMS replicating and glass bonding, requiring the cleanroom facilities and experiences in microfabrication. In addition, the single-layer processing limitation of the photolithography makes it difficult to achieve an intricate 3D microchannel. These problems hinder the further popularization and application of soft lithography in microfluidics industry. With the ability to fabricate the components with arbitrary shapes, 3D printing offers a novel and simple way to fabricate complex microchannels. Microfluidic devices can be produced by directly 3D printing or indirectly PDMS replicating from 3D-printed channel molds [11]. Various 3D printing techniques including fused deposition modelling (FDM) [12], direct ink writing (DIW) [13], stereolithography (SLA) [14], and multijet modelling (MJM) [15] have been employed for directly fabricating the microfluidic devices. Unfortunately, it is difficult for these techniques to achieve a channel width less than 500 µm, and no 3D printing material has been found to be more suitable for microfluidic devices than PDMS [16]. Fabricating microchannel by replicating PDMS from the 3D-printed mold is an effective way to solve the above problems. For examples, channel molds with feature sizes ranging from 10 cm to 250 µm were printed by MJM approach, providing an alternative to the photolithography process in soft lithography [17]. Subsequently, the SLA technique has also been used to create channel molds [18]. To further simplify the workflow, the bonding process can be eliminated by FDM printing the sacrificial material to construct channel mold, which is dissolved after PDMS casting and curing [19,20]. Although having great potential, the use of 3D printed sacrificial mold is still unable to avoid the rough channel surface due to the inherent pancake-stack geometry of 3D printing [21]. Heating the printed mold to reshape the channel into a regular semi-elliptical cross-section has been adopted to smooth the channel surface [22]. Although the material shrinkage has not been found, the heat-treating inevitably causes the change of channel geometrical shape. Using a single thermoplastic filament (without superposition), which is extruded by FDM printer, as the sacrificial channel mold is an ideal way to overcome the above mentioned problems. For instance, the 3D helical microfluidic channels with smooth circular cross-section were fabricated by extruding water-soluble polyvinyl alcohol (PVA) helical filament based on rope coiling effect [23]. The use of single extruded filament as sacrificial channel mold provides several other advantages: (i) the channel section can be easily changed by changing the orifice shapes in the printer nozzles [24], and (ii) the channel size can be flexibly adjusted by controlling the material extrusion amount [25]. Nevertheless, to our best knowledge, the influence of extrusion parameters for fabricating filamentary channel mold has not been elaborately investigated up to now.

In this paper, to fabricate microchannels with different structure and size, the influences of main processing parameters including working distance, extrusion amount and printing speed on the morphologies of the extruded acrylonitrile butadiene styrene (ABS) channel molds were systematically investigated. After conducting the PDMS replicating and ABS dissolving, the different-sized straight microchannels with circular and low-aspect-ratio cross-sections were fabricated by adjusting the extrusion amounts. The sinusoidal, 3D curved and cross-linked curved microchannels with different sizes were then printed along straight path by the combined control of working distance, extrusion amount and printing speed. Finally, the combined microchannel consisting of different structural features along straight path and the complex microchannels with structural features along curved serpentine, rectangular serpentine, and spiral paths were also fabricated. The results show that, fabricating different microchannels by controllably extruding sacrificial molds is a promising supplement to the existing fabrication methods for microfluidic devices.

## 2. Materials and Methods

### 2.1. Microchannel Fabrication

**Figure 1:**
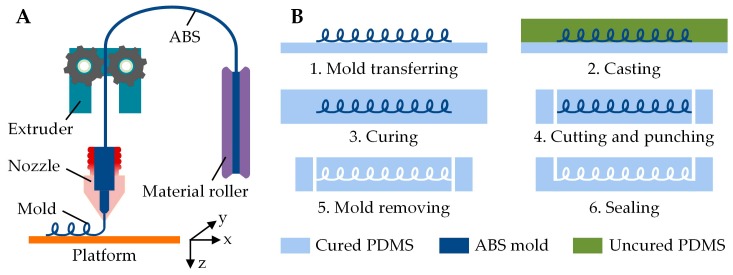
Workflow for fabricating microchannel by using the 3D printed mold-removal method. (**A**) Schematic illustrating the fabrication of microchannel mold by extruding ABS filament with a FDM printer; (**B**) Fabrication process for achieving the microfluidic chip with PDMS replicating and ABS dissolving procedures.

The microchannels were fabricated by using a 3D printed mold-removal method, which has recently been widely employed by researchers [19,24]. The workflow for fabricating microchannel consists of ABS mold printing, PDMS replicating, and sacrificial mold removing, as shown in Figure 1. Firstly, a low-cost and commercially available FDM 3D desktop printer (HOFI X1, Nanjing Baoyan Automation Co., Ltd., Nanjing, China) was used to extrude ABS material at 230 °C through the nozzle with a circular orifice diameter of 245 µm (Figure 1A). The extruded ABS filament was then cooled and solidified on the printing platform (80 °C) to form a microchannel mold. The working distance between nozzle and platform, the extrusion amount at unit printing length, and the moving speed of nozzle are the main processing parameters influencing the structure and size of the printed channel mold, which will be detailly discussed in the results and discussion section. For simplification, in this paper the extrusion amount is used to represent the extrusion amount of ABS material at unit printing length, and the printing speed is used to represent the moving speed of nozzle. After obtaining the channel mold, the PDMS replicating and ABS dissolving procedures were sequentially conducted to achieve a microfluidic chip (Figure 1B). Specifically, the printed mold was transferred onto a 5 mm thick PDMS slab, which was previously cured in a 100 × 100 mm plastic petri dish. A uniform mixture of PDMS prepolymer and curing agent (Sylgard 184, Dow Corning, Midland, MI, USA) with 10:1 mass ratio was degassed and then poured above the channel mold, forming another 5 mm thick PDMS. After curing at 60 °C for 2 h, the PDMS slab was peeled from the petri dish and cut into desired block. Next, the chip-to-world ports with a diameter of 0.5 mm were carefully punched through the two ends of the channel mold. After that, the PDMS block was immersed in acetone and ultrasonically vibrated until all the ABS mold was dissolved, followed by rinsing in deionized water and blow-drying with filtered air. Finally, some uncured PDMS was carefully injected into one half of the punched ports, and then cured for creating an all-PDMS microfluidic chip without any additional bonding process.

### 2.2. Microchannel Characterization

The photos of the fabricated ABS molds were captured by a camera of HUAWEI Mate 20 (Shenzhen, China) with a customized holder, which was printed by the same FDM printer. The customized holder was designed to have two slots for inserting cellphone in both horizontal and vertical directions, therefore the channel molds placed on a glass slide can be viewed from top and side. To ensure the consistency of observation, the distance between the camera and the channel mold was kept at 20 mm. To observe the 3D microstructures of the channel molds, the scanning electron microscopy (SEM) images of the molds were also acquired using a JEOL JSM-7600F scanning electron microscope (Tokyo, Japan). The optical images of the PDMS microchannels were obtained by an inverted microscope (CKX41, Olympus, Tokyo, Japan) with charge-coupled device (CCD) camera (DP27, Olympus, Tokyo, Japan) through the supporting cellSens Entry software. The obtained microscopic images were then processed and analyzed using an open-source ImageJ software (version 1.52a, NIH, Bethesda, MD, USA). For visualization of the fabricated microchannels, a solution of blue dye, prepared at 0.1 wt% by dissolving the erioglaucine disodium salt (Shanghai Jinsui Bio-Technology Co., Ltd., Shanghai, China) into deionized water, was manually perfused into the microfluidic device via PTFE tubings. The optical photos of the microfluidic device were also captured by the camera of HUAWEI Mate 20. A ruler was included in all photos for measurement calibration. To improve clarity, the brightness and contrast of the original channel mold photos were increased by 15% and 20%, while the brightness and contrast of the original microfluidic device photos were both increased by 15%.

## 3. Results and Discussion

### 3.1. Fabrication of Straight Microchannel

**Figure 2:**
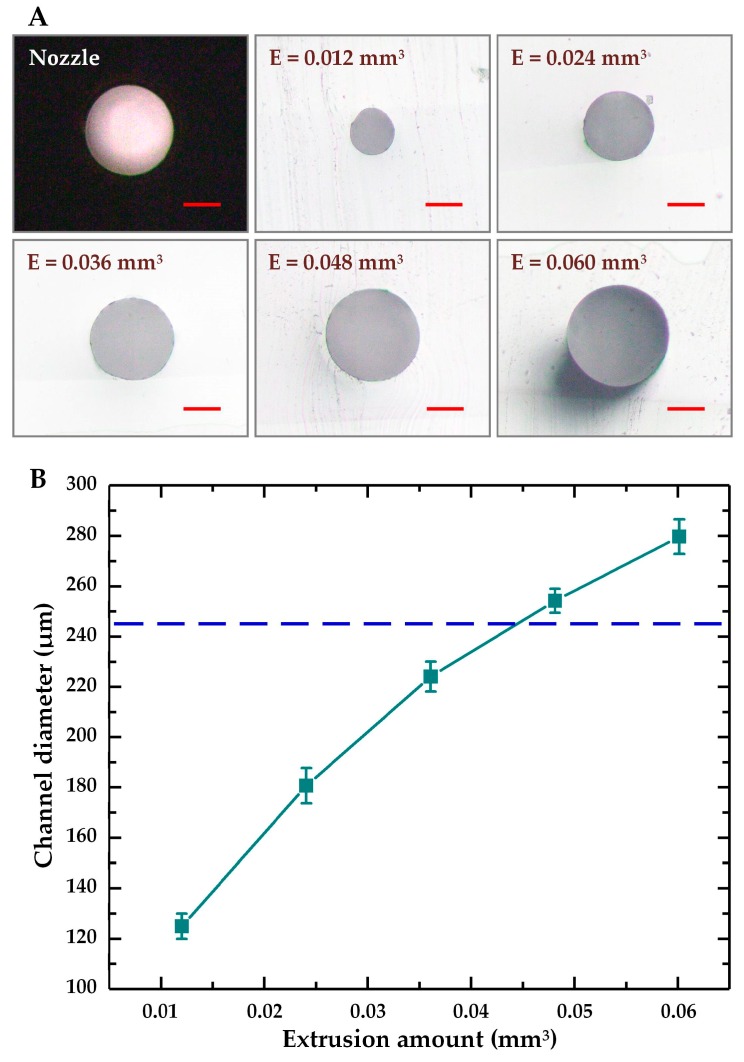
Fabrication of circular straight microchannel with different sizes at low extrusion amounts. (**A**) Cross-sectional images of the nozzle orifice and the microchannels fabricated at different extrusion amounts. The red scale bar is 100 µm; (**B**) Calculated channel diameters at different extrusion amounts. The dotted line indicates the diameter of the nozzle orifice. The error bars represent the standard deviations of diameter differences from ten microchannels.

The straight microchannel can be fabricated by printing the channel mold at low extrusion amount along a straight path. Under such a condition, the molten ABS material is pulled out from the nozzle, so that the printed ABS filament is straightened. For the nozzle with an orifice diameter of 245 µm, the channel molds were printed at different extrusion amounts ranging from 0.012 to 0.06 mm^3^ with an interval of 0.012 mm^3^ (the working distance was fixed at 0.4 mm, and the printing speed was fixed at 200 mm/min). The results show that, the straight channel mold can be obtained when printing ABS material at the extrusion amount of 0.048 mm^3^ or below. However, when the extrusion amount reaches 0.060 mm^3^, the length of the extruded ABS filament becomes greater than the nozzle moving distance, leading to the bending of the extruded channel mold. The cross-sectional images of the microchannels obtained after removal of the ABS molds are shown in Figure 2A. It can be seen that, despite the use of same printing nozzle, the straight microchannels with different sizes can be acquired at different extrusion amounts. Due to the thermoplasticity of ABS plastics, the printed channel molds possess the same cross-section shape as the nozzle orifice, thus the straight microchannels with circular cross-section can be fabricated. The circular microchannel is approximately symmetrical and considered as a good candidate for artificial vessel in biological engineering [26]. The diameters of the fabricated microchannels at different extrusion amounts are calculated and plotted in Figure 2B, the diameter of the nozzle orifice indicated by a dotted line is also included for comparison. It is found that, with the 245 µm diameter nozzle, the circular straight microchannels with diameters ranging from 125 to 280 µm can be obtained by adjusting the extrusion amounts during the channel mold printing.

**Figure 3:**
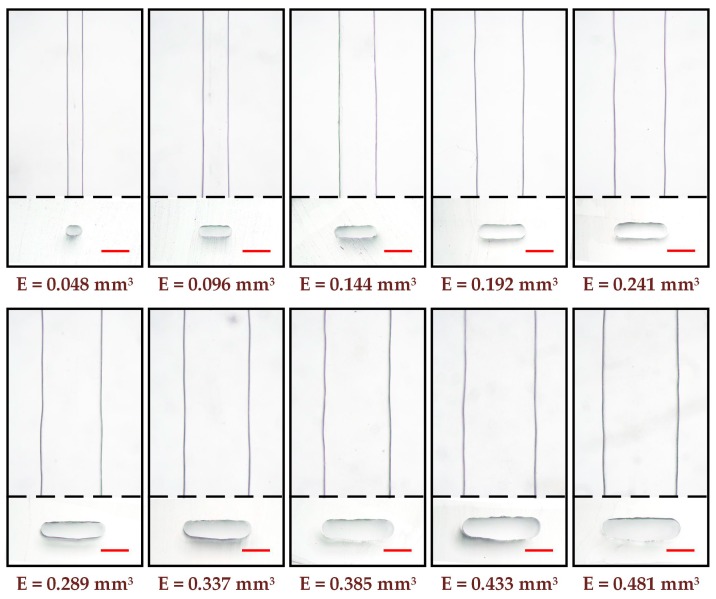
Optical micrographs of the low-aspect-ratio straight microchannels at different extrusion amounts. The top in each group is the vertical view of the microchannel, and the bottom in each group is the cross-sectional view of the microchannel. The red scale bar is 500 µm.

The bending of the extruded filament at high extrusion amount can be eliminated by shortening the working distance between nozzle and platform, so that the molten ABS material is confined to a narrow space to obtain a straight channel mold. To fabricate straight microchannel at high extrusion amount, the working distance was fixed at 0.2 mm, smaller than the diameter of nozzle orifice. The channel molds were printed at different extrusion amounts ranging from 0.048 to 0.481 mm^3^ with an interval of 0.048 mm^3^ (the printing speed was fixed at 200 mm/min). The optical micrographs of the obtained microchannels are shown in Figure 3. The results show that, the low-aspect-ratio straight microchannels with arc sides were fabricated. It is easy to understand the reasons for this phenomenon. During the mold printing, the molten ABS material must be squeezed into the confined space between nozzle and platform. The squeezed ABS needs to swell laterally to release the stress, leading to the bulges near the centers of channel sides where having no boundary constraints. The low-aspect-ratio microchannel has been proved to be an indispensable part for particle manipulation [27]. It can also be seen that, both the width and height of microchannels increase with the increase of extrusion amount, even though the working distance remains the same. This phenomenon suggests that only the lateral swelling is not enough to release the stress within the squeezed ABS, when the channel mold is printed at higher extrusion amount. Thus, the squeezed ABS may also swell upward after the nozzle leaving, leading to the increase in channel height.

### 3.2. Fabrication of Curved Microchannel

**Figure 4:**
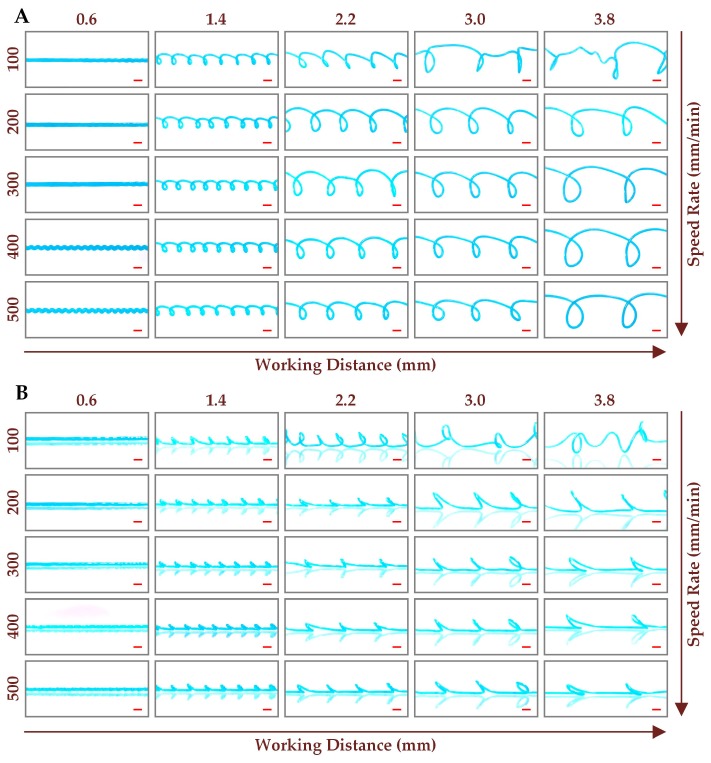
Optical photos of the printed channel molds at different working distances and printing speeds with the extrusion amount fixed at 0.144 mm^3^ from vertical view (**A**) and side view (**B**). The red scale bar represents 1 mm.

In addition to the straight microchannel, the curved microchannels including sinusoidal microchannel, 3D curved microchannel and cross-linked curved microchannel can also be fabricated by the combined control of processing parameters, even though the printing track is still a straight line. To investigate the influences of working distance, extrusion amount and printing speed on the structure and size of microchannels, the channel molds were printed at the working distances ranging from 0.2 to 3.8 mm with an interval of 0.4 mm, the printing speeds ranging from 50 to 500 mm/min with an interval of 50 mm/min, and the extrusion amounts ranging from 0.048 to 0.481 mm^3^ with an interval of 0.048 mm^3^. Figure 4A,B shows the optical photos of the printed channel molds from vertical view and side view respectively, at the specified working distances and printing speeds, while the extrusion amount was fixed at 0.144 mm^3^. It can be clearly seen that, the straight microchannels, sinusoidal microchannels and 3D curved microchannels with different sizes were successfully fabricated. For the working distance of 0.6 mm, the space between nozzle and platform is too small for the molten ABS to form a 3D structure, thus the planar channel molds were printed. It is interesting to find that, the planar channel molds with different structures including straight and sinusoidal can be acquired by adjusting the printing speed. When the printing speed is low, the previously printed ABS filament is likely to be fused with the freshly printed ABS polymer with high temperature, leading to the straight channel molds fabricated at the printing speeds below 350 mm/min. The interaction effect between the previously printed and freshly printed ABS polymers can be reduced by increasing the printing speed. According to the results discussed in the previous section, the length of the extruded ABS filament is greater than the nozzle moving distance with the extrusion amount larger than 0.060 mm^3^. Therefore, with the extrusion amount fixed at 0.144 mm^3^, the printed ABS filament must be bended to accommodate the motion distance of nozzle, leading to the sinusoidal channel molds fabricated at the printing speeds higher than 350 mm/min. The fabricated sinusoidal microchannels can be used as a passive micromixer to improve the mixing performance [28].

Except for the planar microchannels, the 3D curved microchannels, which have been proved to be an effective approach for 3D particle focusing [29], can be also fabricated at the higher working distances. As discussed above, the extruded ABS filament is longer than the nozzle moving distance when the extrusion amount fixed at 0.144 mm^3^. Therefore, during the mold printing process, the extruded ABS filament was coiled to form a 3D curved channel mold with the working distance fixed at 1.4 mm or above. It can be clearly seen from Figure 4 that, the number of the curved loops in channel molds decreases with the increase of working distance, while the size of the curved loops increases with the increase of working distance. The higher working distance allows longer ABS filament to be coiled between nozzle and platform, leading to the formation of larger curved loop. Since the total length of the extruded ABS filament remains unchanged with the extrusion amount fixed at 0.144 mm^3^, the larger curved loops inevitably result in a lower loop number. It can also be seen that, the number and size of the curved loops are almost not affected by the printing speed. However, the periodic 3D curved channel mold can’t be fabricated for the working distance of 3.0 mm or above, when the printing speed was 100 mm/min. Since the extrusion amount was fixed at 0.144 mm^3^, the extrusion rate of ABS material from nozzle is only determined by the printing speed. The low extrusion rate of ABS material at the printing speed of 100 mm/min makes it hard for the extruded ABS filament to touch the platform, when the working distance is longer than 3.0 mm. Therefore, the extruded ABS filament coils freely in the air, failing to form the periodic 3D curved loops. From the side view of the printed channel molds (Figure 4B), we can see that the height of the 3D curved loops decreases with the increase of the printing speed, means that the extruded ABS filament tends to coil near the platform with higher extrusion rates.

**Figure 5:**
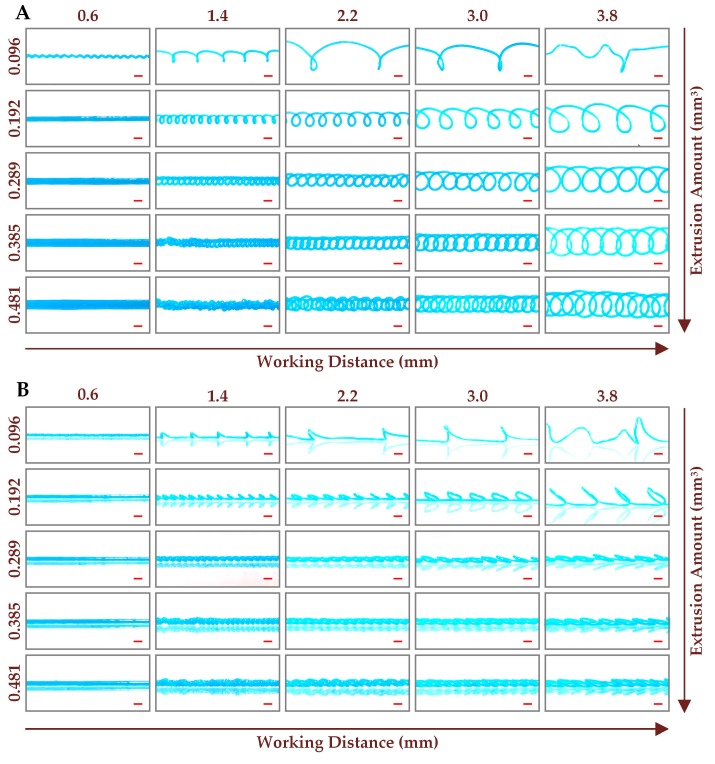
Optical photos of the printed channel molds at different working distances and extrusion amounts with the printing speed fixed at 200 mm/min from vertical view (**A**) and side view (**B**). The red scale bar represents 1 mm.

With the printing speed fixed at 200 mm/min, the optical photos of the printed channel molds at the specified working distances and extrusion amounts are combined in Figure 5. The sinusoidal microchannels, straight microchannels, 3D curved microchannels, and cross-linked curved microchannels with different sizes were successfully fabricated. Since the influence of working distance on the printed mold is previously discussed, here we will focus on the discussion of extrusion amount. For the working distance of 0.6 mm, the planar channel molds with different structures including sinusoidal and straight can be acquired by adjusting the extrusion amount. The sinusoidal channel mold was printed at the extrusion amount of 0.096 mm^3^, while the straight channel molds were printed at the extrusion amount of 0.192 mm^3^ or above. When the extrusion amount is high, the excessive extruded ABS polymer will fill the gap between nozzle and platform during the mold printing process, leading to a straight channel mold with large size. For the working distance of 1.4 mm or above, the 3D curved channel molds and the cross-linked curved channel molds were printed. The results show that, the number of the curved loops in the printed channel molds increases with the increase of extrusion amount. The total length of the extruded ABS filament is extended by increasing the extrusion amount, thus the extruded ABS filament needs to create more curved loops to accommodate the nozzle moving distance. When the extrusion amount is large enough, the adjacent loops will be bonded to each other to form the cross-linked curved microchannels, which can be used for rapid mixing [30]. When the extrusion amount increases further, the periodic curved loops will be destroyed, failing to fabricate the desired microchannel. It can be seen from Figure 5B that, the height of the curved loops in the printed channel molds decreases with the increase of extrusion amount. With the fixed printing speed, the extrusion rate of ABS material from nozzle is only determined by the extrusion amount. Therefore, the extruded ABS filament tends to coil near the platform with the higher extrusion amounts. The low extrusion rate of ABS material at the extrusion amount of 0.096 mm^3^ and the long working distance of 3.8 mm make it hard for the extruded ABS filament to touch the platform, failing to form a desired channel mold.

### 3.3. Fabrication of Complex Microchannel

**Figure 6:**
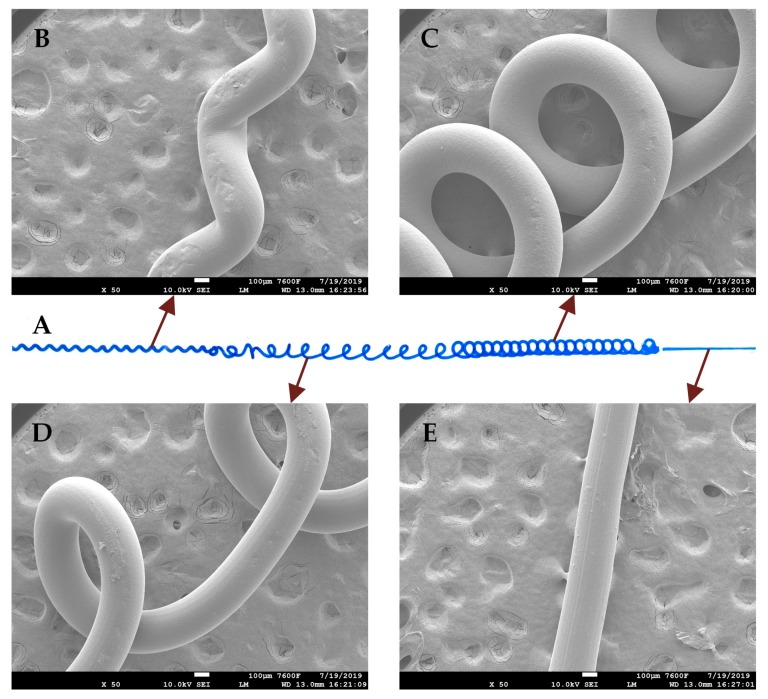
Fabrication of the combined microchannel with different structural features along straight path. (**A**) Optical photo of the fabricated microchannel. A blue dye solution is perfused into the microchannel for visualization; SEM images of the corresponding channel molds for sinusoidal section (**B**), cross-linked curved section (**C**); 3D curved section (**D**) and straight section (**E**).

According to the results discussed above, different microchannels such as straight microchannel, sinusoidal microchannel, 3D curved microchannel and cross-linked curved microchannel with different sizes can be separately fabricated by the combined control of processing parameters including the working distance, extrusion amount and printing speed. Since all the processing parameters can be instantaneously changed during the mold printing process, a combined microchannel consists of all the four kinds of structural features can also be fabricated. Figure 6 shows the photo of the fabricated microchannel perfused with a blue dye solution for visualization, the SEM images for the four different structural features in the channel molds are also included. The straight section in the combined channel mold was printed at the working distance of 0.2 mm, the extrusion amount of 0.048 mm^3^, and the printing speed of 200 mm/min. The sinusoidal section was printed at the working distance of 0.6 mm, the extrusion amount of 0.096 mm^3^, and the printing speed of 500 mm/min. The 3D curved section was printed at the working distance of 1.4 mm, the extrusion amount of 0.192 mm^3^, and the printing speed of 100 mm/min. The cross-linked curved section was printed at the working distance of 1.8 mm, the extrusion amount of 0.385 mm^3^, and the printing speed of 200 mm/min. The results show that, microchannels with different structures and sizes can be arbitrarily combined into a single microfluidic device by controllably extruding the sacrificial molds. The SEM images of the channel molds show that, the whole combined microchannel has a cylindrical surface, despite the channel structure changes.

**Figure 7:**
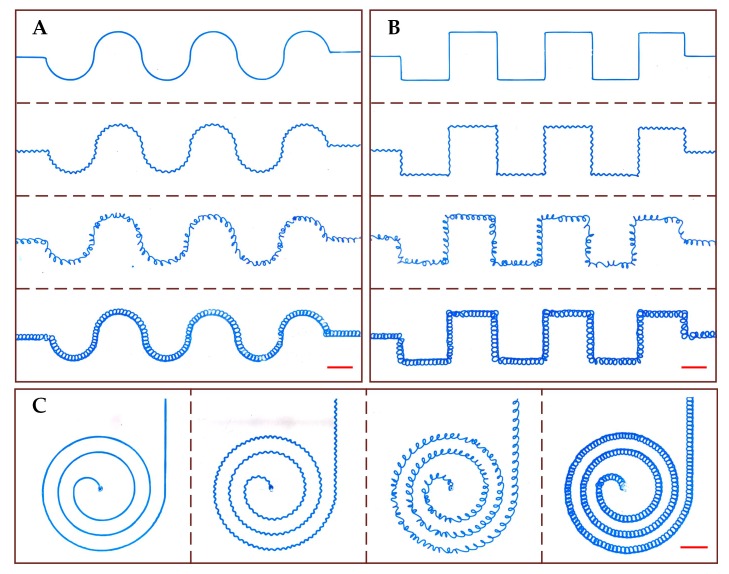
Optical photos of the complex microchannels with different structural features along curved serpentine path (**A**), rectangular serpentine path (**B**), and spiral path (**C**). A blue dye solution is perfused into the microchannel for visualization. The red scale bar represents 5 mm.

In addition to the straight path, the complex microchannels with different structural features were also printed along the curved serpentine, rectangular serpentine, and spiral paths (Figure 7). The processing parameter for printing channel mold with each structural feature is same as mentioned in the previous paragraph. For the curved serpentine path, the diameter of the semicircular unit is 10 mm. For the rectangular serpentine path, the width and height of the rectangular unit are 10 mm and 5 mm, respectively. For the Archimedean spiral path, the initial radius is set at 2.5 mm, and the spacing between two adjacent loops is set at 3 mm. It can be seen form Figure 7 that, the curved serpentine, rectangular serpentine and spiral microchannels with smooth, sinusoidal and cross-linked curved features were all successfully fabricated. However, the periodical 3D curved loops were not formed in the curved serpentine, rectangular serpentine and spiral microchannels. The results indicate that, the 3D curved loops are more susceptible to the nozzle printing trajectory. As can be seen from Figure 4B and Figure 5B, the height of the 3D curved loops is significantly larger than other structures. The 3D curved loops far from the platform are more likely to be affected by the nozzle movement, when printing channel mold along a complex path. This problem can be solved by controlling a multi-degree of freedom (DOF) motion platform to ensure a straight movement of the printing nozzle, even though the microchannel pattern is complex.

## 4. Conclusions

In conclusion, we have explored the fabrication of microchannels with different structure and size by controllably extruding sacrificial molds. The influences of the main processing parameters including working distance, extrusion amount and printing speed on the printed ABS molds were systematically investigated. By conducting the PDMS replicating and ABS dissolving, the all-PDMS microchannels could be easily produced without the complex bonding process. The circular and low-aspect-ratio straight microchannels with different sizes were firstly fabricated by adjusting the extrusion amounts. Then the sinusoidal, 3D curved and cross-linked curved microchannels along the straight path were fabricated, either independently or in combination, by the combined control of working distance, extrusion amount and printing speed. At last, the complex microchannels with different structural features were also printed along the curved serpentine, rectangular serpentine, and spiral paths. It should be noted that, the cross-sectional shape of the complex microchannel can be further changed by modifying the orifice pattern in the printer nozzle.

## References

[1] Whitesides G.M. (2006). The origins and the future of microfluidics. Nature.

[2] Rothbauer M., Zirath H., Ertl P. (2018). Recent advances in microfluidic technologies for cell-to-cell interaction studies. Lab Chip.

[3] Gorkin R., Park J., Siegrist J., Amasia M., Lee B.S., Park J.M., Kim J., Kim H., Madou M., Cho Y.K. (2010). Centrifugal microfluidics for biomedical applications. Lab Chip.

[4] Elvira K.S., Solvas X.C.I., Wootton R.C.R., deMello A.J. (2013). The past, present and potential for microfluidic reactor technology in chemical synthesis. Nat. Chem..

[5] Meredith N.A., Quinn C., Cate D.M., Reilly T.H., Volckens J., Henry C.S. (2016). Paper-based analytical devices for environmental analysis. Analyst.

[6] Chen W., Lam R.H.W., Fu J. (2012). Photolithographic surface micromachining of polydimethylsiloxane (PDMS). Lab Chip.

[7] Deng Y., Yi P., Peng L., Lai X., Lin Z. (2014). Experimental investigation on the large-area fabrication of micro-pyramid arrays by roll-to-roll hot embossing on PVC film. J. Micromech. Microeng..

[8] Hong T.-F., Ju W.-J., Wu M.-C., Tai C.-H., Tsai C.-H., Fu L.-M. (2010). Rapid prototyping of PMMA microfluidic chips utilizing a CO_2_ laser. Microfluid. Nanofluid..

[9] Tian F., Cai L., Chang J., Li S., Liu C., Li T., Sun J. (2018). Label-free isolation of rare tumor cells from untreated whole blood by interfacial viscoelastic microfluidics. Lab Chip.

[10] Duffy D.C., McDonald J.C., Schueller O.J.A., Whitesides G.M. (1998). Rapid prototyping of microfluidic systems in poly(dimethylsiloxane). Anal. Chem..

[11] Sochol R.D., Sweet E., Glick C.C., Wu S.-Y., Yang C., Restaino M., Lin L. (2018). 3D printed microfluidics and microelectronics. Microelectron. Eng..

[12] Pranzo D., Larizza P., Filippini D., Percoco G. (2018). Extrusion-based 3D printing of microfluidic devices for chemical and biomedical applications: A topical review. Micromachines.

[13] Therriault D., White S.R., Lewis J.A. (2003). Chaotic mixing in three-dimensional microvascular networks fabricated by direct-write assembly. Nat. Mater..

[14] Bhargava K.C., Thompson B., Malmstadt N. (2014). Discrete elements for 3D microfluidics. Proc. Natl. Acad. Sci. USA.

[15] Sochol R.D., Sweet E., Glick C.C., Venkatesh S., Avetisyan A., Ekman K.F., Raulinaitis A., Tsai A., Wienkers A., Korner K. (2016). 3D printed microfluidic circuitry via multijet-based additive manufacturing. Lab Chip.

[16] Mohamed M.G.A., Kumar H., Wang Z., Martin N., Mills B., Kim K. (2019). Rapid and inexpensive fabrication of multi-depth microfluidic device using high-resolution LCD stereolithographic 3D printing. J. Manuf. Mater. Process..

[17] McDonald J.C., Chabinyc M.L., Metallo S.J., Anderson J.R., Stroock A.D., Whitesides G.M. (2002). Prototyping of microfluidic devices in poly(dimethylsiloxane) using solid-object printing. Anal. Chem..

[18] Comina G., Suska A., Filippini D. (2014). Low cost lab-on-a-chip prototyping with a consumer grade 3D printer. Lab Chip.

[19] Saggiomo V., Velders A.H. (2015). Simple 3D printed scaffold-removal method for the fabrication of intricate microfluidic devices. Adv. Sci..

[20] Goh W.H., Hashimoto M. (2018). Dual sacrificial molding: Fabricating 3D microchannels with overhang and helical features. Micromachines.

[21] Goh W.H., Hashimoto M. (2018). Fabrication of 3D microfluidic channels and in-channel features using 3D printed, water-soluble sacrificial mold. Macromol. Mater. Eng..

[22] Dahlberg T., Stangner T., Zhang H., Wiklund K., Lundberg P., Edman L., Andersson M. (2018). 3D printed water-soluble scaffolds for rapid production of PDMS micro-fluidic flow chambers. Sci. Rep..

[23] Yang W., Zhu T., Jin Y., Fu J. (2017). Facile fabrication of helical microfluidic channel based on rope coiling effect. Microsyst. Technol..

[24] Tang W., Fan N., Yang J., Li Z., Zhu L., Jiang D., Shi J., Xiang N. (2019). Elasto-inertial particle focusing in 3D-printed microchannels with unconventional cross sections. Microfluid. Nanofluid..

[25] Tang W., Fan N., Li Z., Xiang N., Yang J. (2019). Facile fabrication of microchannel with unconventional cross-section using 3D printed sacrificial mould. Chin. J. Anal. Chem..

[26] Choi C.-H., Yi H., Hwang S., Weitz D.A., Lee C.-S. (2011). Microfluidic fabrication of complex-shaped microfibers by liquid template-aided multiphase microflow. Lab Chip.

[27] Zhang J., Yan S., Yuan D., Alici G., Nguyen N.T., Warkiani M.E., Li W.H. (2016). Fundamentals and applications of inertial microfluidics: A review. Lab Chip.

[28] Özkan A., Erdem E.Y. (2015). Numerical analysis of mixing performance in sinusoidal microchannels based on particle motion in droplets. Microfluid. Nanofluid..

[29] Paiè P., Bragheri F., Di Carlo D., Osellame R. (2017). Particle focusing by 3D inertial microfluidics. Microsyst. Nanoeng..

[30] Liu K., Yang Q., Chen F., Zhao Y., Meng X., Shan C., Li Y. (2015). Design and analysis of the cross-linked dual helical micromixer for rapid mixing at low Reynolds numbers. Microfluid. Nanofluid..

